# In Situ Growth of Silver Nanoparticles on Chitosan Matrix for the Synthesis of Hybrid Electrospun Fibers: Analysis of Microstructural and Mechanical Properties

**DOI:** 10.3390/polym14040674

**Published:** 2022-02-10

**Authors:** Karina Santiago-Castillo, Aidé Minerva Torres-Huerta, Deyanira del Ángel-López, Miguel Antonio Domínguez-Crespo, Héctor Dorantes-Rosales, Diana Palma-Ramírez, Helen Willcock

**Affiliations:** 1 CIAMS, CICATA-Altamira, Instituto Politécnico Nacional, Km. 14.5 Carretera Tampico-Puerto Industrial Altamira, Altamira 89600, Mexico; k.sgcast@outlook.com; 2 Departamento de Materiales Nanoestructurados, Unidad Profesional Interdisciplinaria de Ingeniería campus Hidalgo (UPIIH), Instituto Politécnico Nacional, Km. 1 + 500, Carretera Pachuca-Actopan, San Agustín Tlaxiaca 42162, Mexico; 3 Escuela de Ingeniería y Ciencias, Instituto Tecnológico y de Estudios Superiores de Monterrey, Ave. Eugenio Garza Sada 2501, Monterrey 64849, Mexico; yaderani@hotmail.com; 4 Área de ciencias químicas, exactas y tecnológicas, Universidad del Noreste, Prolongación Av. Hidalgo 6315 Col Nuevo Aeropuerto, Tampico 89337, Mexico; 5 Departamento de Metalurgia, ESIQIE, Instituto Politécnico Nacional, Ciudad de México 07300, Mexico; hectordorantes@yahoo.com; 6 Centro Mexicano para la Producción más Limpia (CMPL), Instituto Politécnico Nacional, Av. Acueducto s/n, La Laguna Ticomán, México City 07340, Mexico; d.palma.rmz@gmail.com; 7 Department of Materials, Loughborough University, Loughborough LE11 3TU, UK; H.willcock2@lboro.ac.uk

**Keywords:** chitosan, silver nanoparticles, polyvinyl alcohol, electrospun fibers, mechanical properties

## Abstract

A viable alternative for the next generation of wound dressings is the preparation of electrospun fibers from biodegradable polymers in combination with inorganic nanoparticles. A poly(vinyl alcohol)-chitosan-silver nanoparticles (PVA-CTS-Ag NPs) system has been developed for antimicrobial and wound healing applications. Here, the preparation of PVA-CTS-Ag electrospun fibers using a two-step process is reported in order to analyze changes in the microstructural, mechanical, and antibacterial properties and confirm their potential application in the biomedical field. The Ag nanoparticles were well-dispersed into the chitosan matrix and their cubic structure after the electrospinning process was also retained. The Ag NPs displayed an average diameter of ~33 nm into the CTS matrix, while the size increased up to 213 nm in the PVA-CTS-Ag(NPs) fibers. It was observed that strong chemical interactions exist between organic (CTS) and inorganic phases through nitrogenous groups and the oxygen of the glycosidic bonds. A defect-free morphology was obtained in the PVA-CTS-Ag NPs final fibers with an important enhancement of the mechanical properties as well as of the antibacterial activity compared with pure PVA-CTS electrospun fibers. The results of antibacterial activity against *E. coli* and *S. aureus* confirmed that PVA-CTS-Ag(NPs) fibers can be potentially used as a material for biomedical applications.

## 1. Introduction

In recent years, nanofibers or filaments, of sizes smaller than 100 nm in diameter, have attracted the attention of researchers for the production of advanced materials with applications in catalysis, tissue engineering, textiles, membranes, electronics, optics, sensors, coatings, biomedicine and biotechnology [1,2]. Currently, there are different techniques to produce micro- and nanofibers, such as interfacial polymerization, phase separation, replica synthesis and electrospinning. Electrospinning is one of the most widely used techniques to produce various materials due to its versatility, low cost, reproducibility and scalability [3]. It is an electrohydrodynamic technique for the fabrication of micro- or nanometric fibers using melted polymers or polymeric solutions [4]. Nanofibers prepared by electrospinning are characterized by a large surface area and, in some cases, pores can be formed on their surface [5]. They can be synthesized from natural or synthetic polymers, depending on their application. To carry out the processing, the application of an electric field to a conductive solution is required to cause elongation of the material and thus to produce filaments, which are collected uniaxially or coaxially on a metallic device [6]. When using the uniaxial operation, only one capillary is used, while in the coaxial mode, one capillary contains the polymeric solution and another capillary contains a compound material, which allows for the preparation of core-shell type electrospun nanofibers, a specific type of microstructure in polymers [7,8]. To date, electrospun fibers have been prepared from more than 100 synthetic and natural polymers [2]. Some of the most used synthetic polymers by electrospinning are nylon 6,6, poly (ethylene oxide) (PEO), poly(vinyl alcohol) (PVA), poly (urethane) (PU) and poly(methyl methacrylate) (PMMA) [9]. On the other hand, poly (lactic acid) (PLA), collagen and chitosan (CTS), are biodegradable polymers, respectively, that have been used for electrospun fiber manufacture and, among them, CTS stands out due to its antiseptic properties [10,11,12]. CTS, whose chemical name is poly [β-(1-4)-2-amino-2-deoxy-D-glucopyranose], is obtained from the chemical modification of chitin [13]. Chitin is the second-most abundant polysaccharide in nature after cellulose, and is present in the shells of crustaceans, fungi and insects [14]. CTS can contain between 6% and 9% nitrogen and comes in different degrees of purity, molecular weight and physicochemical properties (odor, color, solubility, viscosity and reactivity) [15]. It is a biodegradable, biocompatible, non-toxic polymer [16], with antibacterial, antifungal and antioxidant properties [17,18]; it participates during the regulation of blood coagulation and it helps for the acceleration of wound healing [19]. It has applications in agriculture [20], food [21], water treatment [22], cosmetics [23] and biomedicine [24]. CTS is a biopolymer whose pH varies from neutral to slightly basic; however, in acidic solutions its amino groups are protonated ($-{\mathrm{NH}}_{3}^{+}$), modifying its physical properties (polarity, solubility and conductivity) [25]. Chitosan has some disadvantages, such as its polycationic nature, rigid chemical structure and specific inter- and intramolecular interactions that make chitosan solutions highly viscous, which makes the direct electrospinning of chitosan solutions difficult to control [26]. Therefore, it is combined with other natural or synthetic polymers to improve its mechanical properties and processability; such as PVA and PEO [27,28]. 

PVA is a synthetic polymer, obtained by the hydrolysis (alcoholysis) of poly(vinyl acetate), (C_4_H_6_O_2_)_n_ [29]. The degree of hydrolysis of PVA is an important property when used for membrane applications, mainly because of crystallinity and polarity. The higher the degree of hydrolysis, the higher the degree of crystallinity, which is generated due to the presence of the polar hydroxyl groups (OH^–^) facilitating the organization of crystals while modifying the selectivity [30]. PVA is a highly flexible biodegradable polymer, biocompatible, non-toxic, hydrophilic and water soluble, at temperatures above 80 °C [31,32,33]. PVA has been reported to improve the mechanical properties, biodegradability, biocompatibility, hydrophilicity and processability of other polymers to prepare electrospun fibers. An example of this would be when it is blended with CTS [34]. In addition, PVA improves the viability, proliferation and gene expression of fibroblasts and therefore the biocompatibility of electrospun fibers [35]. Thus, it has been used in the biomedical field as a wound dressing material, drug delivery system, in contact lenses and as a prosthesis, mainly due to its affinity for blood, plasma and live tissues [33,36,37]. 

Electrospun fibers from biodegradable and biocompatible natural polymers, such as CTS and PVA, have great potential in the biomedical field due to the synergy among properties, i.e., low toxicity, high porosity, light weight, interconnectivity between pores and high surface area. In addition, the large surface area/volume ratio allows its specific surface area in contact with the media in which it is found to be very active, having a porous structure that favors cell adhesion, proliferation and differentiation [38,39]. Electrospun nanofibers of PVA-CTS are effective in biomedical applications, which includes bandages or dressings for wound treatment, diabetic foot ulcers, tissue engineering and drug delivery [40,41,42]. 

In recent years, it has been shown that the incorporation of pure or ceramic NPs such as Ag [43,44], TiO_2_ [45], CeO_2_ [46,47] and ZnO [48,49,50] into natural polymer matrices, during the processing of hybrid natural nanofibers, can improve the wound healing process and be used as dressing materials. In particular, it has been established that the addition of Cu NPs into the PVA-CTS system improves the rate of wound closure. In this case, Cu NPs stimulate keratinocyte and fibroblast proliferation, collagen synthesis, and re-epithelialization, which are key factors for wound healing. Unfortunately, a major drawback in the system is the toxicity of the copper nanoparticles that affect cells in the liver and kidneys. Additionally, they can be genotoxic and capable of producing oxidative stress to the cells [51]. Mohandas and coworkers reported that other nanostructures such as gold exhibit significantly lower toxicity in the PVA-CTS system [52]. Similar works exist that show that the PVA-CTS-Au NPs system combined with *pomegranate extract (Punica granatum, PE)* improved the bactericidal activity, mechanical properties, and the long-term stability of the electrospun fibers; however, a major disadvantage is the cost of production [53].

On the other hand, Ag NPs have been shown to have antibacterial, antifungal and antiviral properties [54]. In addition, Ag NPs possess anti-inflammatory properties during wound healing, so they have been widely used in the biomedical field, particularly in drug delivery, medical mask coatings, wound treatment dressings, bone implants and prostheses [43,55]. As a consequence, there are several methods for their synthesis, and from these, one of the most studied approaches is through chemical reduction [44]. Thus, the combination of PVA-CTS with Ag NPs obtaining hybrid natural electrospun fibers can be used as membranes to improve the microbicidal properties, the collagen deposition on the wound area as well as to diminish the inflammatory process and to favor the healing of wounds and burns [56]. In this context, novel PVA-CTS with Ag NPs hybrid materials have been reported that are made via the electrospinning method, which improves workability and antibacterial ability, and which in turn is able to be applied as coating or wound dressing material [57]. Unfortunately, the authors did not show a detail of analysis about the addition of those nanoparticles on the microstructure and mechanical properties. 

Based on previous developments, the goal of this work was carried out the in situ synthesis of Ag NPs into a solution of CTS blended with high molecular weight PVA for the production of electrospun fibers in order to analyze changes on the microstructural, mechanical, antibacterial properties and to confirm the potential application for the treatment of wounds and/or burns. In addition, the results are compared with a similar system previously reported, PVA-CTS-ZnO NPs, to evaluate changes on these properties depending upon the nanoparticle-type [49]. 

## 2. Materials and Methods

### 2.1. Reagents and Materials

In this work, the reagents were used without further purification. CTS (molecular weight 190–310 kDa, 75–85% degree of deacetylation and 90% purity), silver nitrate salt (AgNO_3_, 99% purity) and D-(+)-glucose (C_6_H_12_O_6_, 99% purity) were purchased from Sigma Aldrich^®^ (St. Louis, MO, USA); glacial acetic acid (C_2_O_2_H_4_, 99.8% purity) was purchased from Fermont^®^ (Monterrey, N.L., México). High molecular weight PVA (146–186 kDa, 99.3% purity +99% hydrolyzed) purchased from Sigma Aldrich^®^ (St. Louis, MO, USA) was used. Bacterial strains *Escherichia coli* (*E. coli*) *ATCC 25922 and Staphylococcus aureus (S. aureus) ATCC 25923* were used. Mueller Hinton agar was purchased from BD Bioxon (Franklin Lakes, NJ, USA).

### 2.2. CTS-Ag NPs In Situ Synthesis

The synthesis of CTS-Ag NPs was carried out by the chemical reduction method with glucose according to the procedure described by Abdelgawad et al. using silver nitrate as the metal precursor [57]. A 2.1 M solution of silver nitrate and a 5.6 M solution of glucose were prepared. On the other hand, CTS (2 g) was dissolved in 98 mL of 2 wt% acetic acid to obtain a 2 wt% CTS solution. The CTS solution was placed into a three-necked flask, which was connected to a reflux system using an oil bath. The system was brought to 95 °C maintaining magnetic stirring. When the temperature was reached, 2 mL of silver nitrate solution was added into it and, subsequently, 3 mL of the glucose solution was added using a flow rate of 1.5 mL/h. The reaction time was 6 h, maintaining the temperature at 95 °C with magnetic stirring. After the reaction, the flask with the resulting colloidal solution was allowed to cool down and stored in an amber flask at 4 °C. The final composition of the CTS:Ag NPs compound was computed considering that 2 mL of AgNO_3_ solution (2.1 M) was used. The concentration was equivalent to 0.72 g (AgNO_3_) that corresponds to 0.4571 g of metallic silver, which was considered as the theoretical amount of Ag nanoparticles that can be obtained for each 100 mL of CTS:Ag NPs. Thus, the final ratio of CTS:Ag NPs compound was about 99.5429:0.4571. 

### 2.3. Preparation of PVA-CTS and PVA-CTS-Ag NPs Electrospun Fibers

The electrospun fibers mats were prepared using as a reference the conditions reported in a previous work by the research team in which PVA-CTS- ZnO NPs electrospun fibers were produced [49]. An 8 wt% PVA solution was prepared in distilled water at 90 °C, which was stirred for 2 h and a 2 wt% CTS solution was prepared into a 2 wt% acetic acid solution. Both solutions were blended to obtain a PVA-CTS ratio, 60–40 wt%. This ratio was selected based on previous reports, where it was demonstrated to be an optimal yield for the synthesis of PVA-CTS electrospun fibers [57]. The solution was kept under stirring for 1 h. From this blend, the electrospun fibers were synthetized. 

PVA-CTS-NPs Ag compound fibers were performed using the following procedure: 4 g of the colloidal CTS-Ag NPs solution were blended with 6 g of the 8 wt% PVA solution to keep a 60–40 ratio. The individual solutions of CTS and PVA were magnetically stirred for at least 2 h up to make them homogeneous. Thereafter, the blend was kept under magnetic stirring for 20 min before using it [58]. To compare the properties of the fibers without CTS and without nanoparticles, pure 8 wt% PVA fibers were also prepared. 

**Figure 1:**
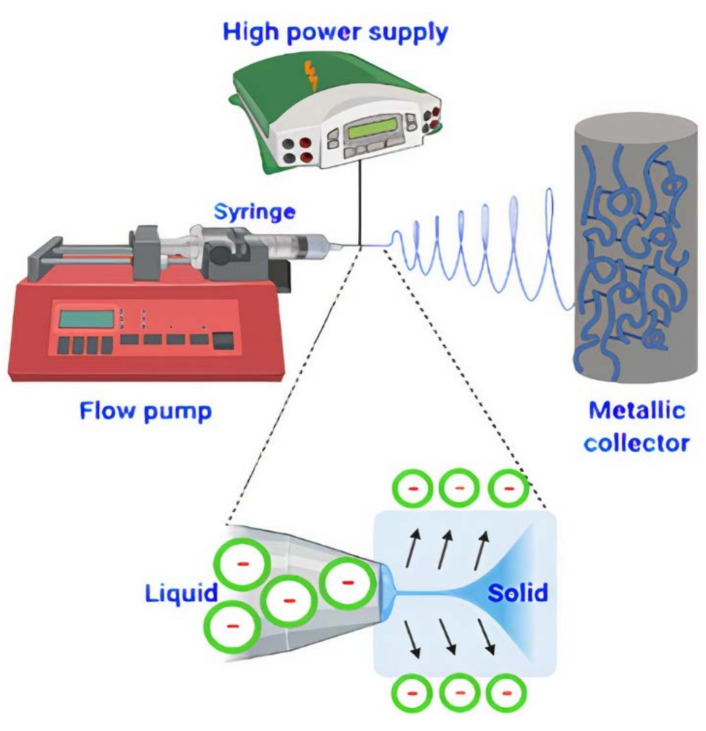
Scheme of general outline of electrospinning process in which the Taylor’s cone and flow charge interactions due to electric field applied are shown.

The electrospun fiber mats were prepared using MTI CORP electrospinning equipment, model MSK-NFES-4LD (Richmond, CA, USA). Each sample was added in a 10 mL capacity plastic syringe, connected to a 22 G hypodermic needle (0.7 mm inner diameter). The metal tip was connected to a high voltage supply. The solution flow rate was 0.18 mL/h. Samples were collected on an aluminum foil (0.2 mm thickness) wrapped around the rotating cylindrical collector with a diameter of 20 cm. The electric potential of 25 kV was applied while the distance between the needle and the collector was 20 cm. A typical electrospinning equipment configuration is shown in the following scheme (Figure 1).

### 2.4. Characterization of Nanostructures and Electrospun Fibers

The pure CTS and CTS-Ag NPs samples were optically and structurally characterized by Fourier Transform Infrared (FTIR) spectroscopy, X-Ray diffraction (XRD), ultraviolet-visible (UV-Vis) spectroscopy and dynamic light scattering (DLS), respectively; while the electrospun PVA-CTS and PVA-CTS-Ag NPs fibers were characterized by FTIR, morphologically by scanning electron microscopy (SEM) and transmission electron microscopy (TEM), and their mechanical properties were analyzed by nanoindentation. Additionally, the antibacterial activity of the electrospun fibers was assessed by the Kirby Bauer method (disc diffusion method). 

FTIR measurements were performed to determine the interaction between the polymeric matrix and NPs. The pure CTS (solid) analysis was carried out through pellet method with potassium bromide (KBr) using a 1:100 ratio. On the other side, for analysis of the CTS-Ag NPs compound, films were obtained from colloidal solutions and analyzed without any additional preparation using the ATR mode. The electrospun fibers were analyzed directly, without previous preparation, using an air blank. For analysis, a Perkin Elmer spectrophotometer, model Spectrum One, was used (Waltham, MA, USA). Measurements were carried out in a frequency range of 4000–400 cm^−1^ with a resolution of 2 cm^−1^. The structural analysis of the samples was carried out using a diffractometer Bruker D8 Advance (Billerica, MA, USA), which operates with Bragg-Brentano geometry (θ–2θ), voltage 40 kV, current 40 μA and a Kα radiation of Cu (λ = 1.5406 Å) in a 2θ range from 10° to 90°. UV-Vis analysis was performed to confirm the formation of the nanoparticles into the CTS matrix. For this purpose, an Agilent Technologies spectrophotometer was used, series Cary 5000 (Santa Clara, CA, USA), in absorbance mode for CTS-Ag NPs compound (keeping constant volume). The analysis was carried out in a wavelength range of 200–800 nm.

The mean particle size (hydrodynamic radius) and polydispersity index (PDI) of CTS-Ag NPs solution colloids were measured using a Delsa Nano system (Beckman Coulter, UK). Measurement parameters were as follows: a laser wavelength of 658 nm and a medium refractive index of 1.3328. Before DLS measurement, the colloid was dissolved in 3 mL of deionized water and passed through a 0.22 μm polyethersulfone (PES) membrane. The sample was loaded into disposable microcuvette, and five measurements were performed, for which the mean result was recorded.

The morphology of the nanocomposite fibers was observed using a Carl Zeiss Field Emission Gun Scanning Electron Microscopy (FEG-SEM), model (LEO) 1530 VP (Jena, Germany). The samples were fitted on a conductive carbon ribbon and coated with Au/Pd using an Emitech SC7640 cathodic coating device, for 90 s. The microstructure and the dispersion of the inorganic NPs in the synthetized samples were evaluated through TEM. The samples were collected during the electrospinning process by placing a grid close to a rotating collector. The samples were analyzed using a JEOL-2000 FX-II microscope (Tokyo, Japan), operating at an accelerating voltage of 200 kV. The hardness and elastic modulus of electrospun fibers were evaluated through nanoindentation. NHT3 nanoindentation equipment, brand Anton Paar (Graz, Austria), Berkovich-type pyramidal geometry diamond indenter was used, operating at a load of 5 mN. The sample mats were discs of 3 cm of diameter. 

The antibacterial assessment was carried out through the disc diffusion technique (Kirby Bauer). The bacterial strains *E coli ATCC (American Type Culture Collection) 25922 and S. aureus ATCC 25923*, common bacteria in wounds, were used. Separately, colonies of *E. coli* (Gram negative) and *S. aureus* (Gram positive) were cultivated on a Mueller-Hinton agar medium. Bacteria were swabbed uniformly onto the petri dish using sterile cotton swabs. Subsequently, the 2 cm diameter nanofiber mat discs were placed on a Mueller Hinton agar medium containing bacterial culture. Finally, the plates were incubated at 37 °C for 24 h. For each sample, the inhibition halo was measured in millimeters (mm) using a ruler and compared with the control (a culture not exposed to the fibers) for each strain. Three replicates were carried out under the same conditions for each sample.

## 3. Results

### 3.1. Characterization of the CTS-Ag NPs Compounds

Chemical, Structural and Optical Properties

**Figure 2:**
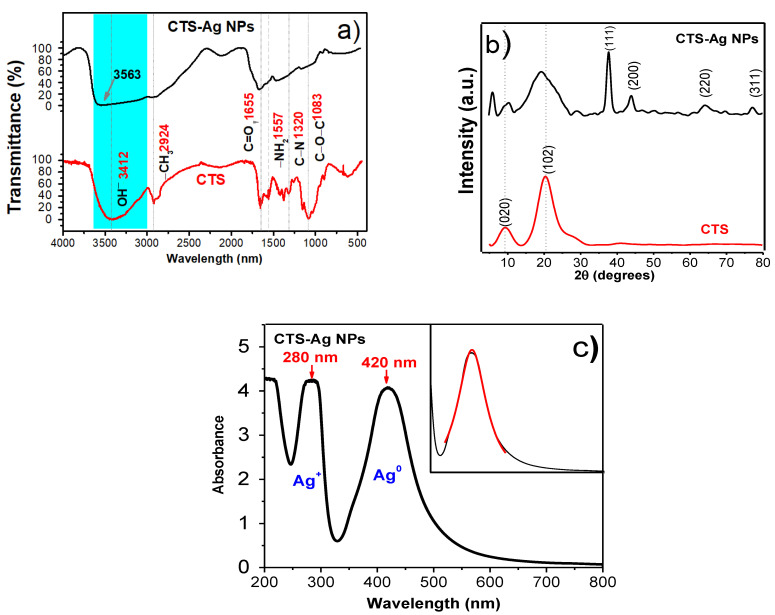
(**a**) FTIR of pure chitosan and the CTS Ag NPs compound, (**b**) X Ray Diffraction pattern of pure chitosan and the CTS-Ag NPs compound and (**c**) UV-Vis spectrum of the CTS-Ag NPs compound showing the absorption band corresponding to the superficial plasmon resonance (SPR) phenomenon of nanometric silver.

The FTIR spectra corresponding to the pure CTS and the CTS-Ag NPs compounds are shown in Figure 2a. The CTS spectrum revealed the characteristic overlapping vibration signals of the hydroxyl (–OH^−^), amino (−NH_2_) and amide (−CONH_2_) functional groups at 3412 cm^−1^. Also, bands of the asymmetric stretching of the methyl groups (−CH_3_) were observed at 2924 cm^−1^, the stretching of the carbonyl group (C=O) at 1655 cm^−1^, the band of −CONH_2_ at 1557 cm^−1^ [13], the secondary stretching of the OH^−^ group at 1378 cm^−1^, the C-N stretching of type III amides at 1320 cm^−1^ and the stretching of the glycosidic bond (C−O−C) at 1083 cm^−1^ [25,30]. The broad band between 3500 and 3200 cm^−1^ is characteristic of the stretching of the −NH_2_ groups overlapped with OH^−^ groups [57]. In comparison with our previous study, where ZnO ceramic NPs (CTS-ZnO NPs) were added [49], a significant widening and shifting towards higher wavenumbers was observed at 3563 cm^−1^ (red shift). The displacement suggested an interaction between NH_2_ groups of CTS and Ag NPs. Additionally, an important decrease of the intensity bands corresponding to the asymmetric stretching of the −CH groups (2916 cm^−1^ in CTS) is observed in the spectra, which is attributed to the physical interactions between these groups and Ag NPs. The most significant changes between both spectra are correlated with the nitrogenous groups. The bands of amino, acetamide groups and the C−N vibration, initially observed at 1557 cm^−1^, 1378 cm^−1^ and 1320 cm^−1^, respectively, disappear after adding the Ag NPs, confirming a strong organic-inorganic interaction. The broadening of the glycosidic bond stretching band (1083 cm^−1^) was observed, which confirms interactions between Ag NPs and the oxygen atoms of glycosidic bonds, C−O−C, that are linked at each monomeric unit. This interaction has been previously established as the interaction of covered Ag NPs by chitosan [59]. Other modifications are observed in the band at 1655 cm^−1^, characteristic of the carbonyl stretching of the N-acetyl group, which becomes broad.

The structural properties of CTS and the CTS-Ag NPs compounds were also studied by XRD measurements. The diffraction pattern of pure CTS (Figure 2b) shows the two characteristic reflections of the polysaccharides compounds, at 9.2° and 20.3° (2θ) that correspond to the α and γ phases, respectively, (crystallographic PDF #00-039-1897 chart) [60]. It can also be observed that the signal at ~13° (2θ) is commonly correlated with the (020) plane of orthorhombic structure [61]. On the other hand, the addition of Ag NPs to form hybrid compounds provokes displacement in the main reflections of CTS which are observed at 10.4° and 19.3°, confirming a strong interaction between organic-inorganic compounds. The crystallographic planes (111), (200) and (311) and crystallographic planes at 38°, 44°, 64° and 77°, respectively, can also be observed, which matches well with the PDF #01-089-3722 chart of the silver cubic structure [61,62]. An initial approximation of the crystallite size for Ag NPs was computed from Scherrer´s equation [63,64]:

(1) $$d=\frac{0.9\lambda }{\beta \cos\theta }$$

The obtained results showed that the Ag NPs present a crystallite size of 14.0 ± 4.0 nm. This approximation is similar to those studies reported for synthesis of Ag NPs using chemical reduction method [65]. The effect of the metallic nanostructures was also analyzed from crystallinity percentage, $Cr_{I}$ (%). The crystallinity percentage of CTS was calculated using the (020) and (102) planes through the following formula:

(2) $$Cr_{I}\left(\%\right)=\left[\frac{I_{020\;\mathrm{or}\;102\;}-I_{\mathrm{am}}}{I_{020\;\mathrm{or}\;102}}\right]\;\times 100$$

In this equation, $Cr_{I}$ (%) is the percentage crystallinity of the sample, *I*_020 or 102_ is the maximum intensity of the reflection corresponding to the (020) or (102) diffraction plane and *I*_am_ is the intensity of the diffraction reflections from the amorphous zone, i.e., 2*θ* = 12.5°. The $Cr_{I}$ (%) of the pure CTS was 78.7%, while the $Cr_{I}$ (%) of the CTS-Ag NPs compound was 63.7%, establishing a reduction of the crystalline order of the CTS during the hybrid compound formation. This value is lower than the reported assessment for the CTS-ZnO NPs compound ($Cr_{I}$ (%) = 86); which is correlated with a stronger interaction between Ag NPs-CTS; i.e., Ag NPs affects the structural order.

The alterations of the optical properties were analyzed by UV-Vis absorption spectra (Figure 2c). In this figure, it can be seen two strong absorption bands located at ~280 nm and ~420 nm, which have been correlated with Ag(0) [66,67,68]. It is evident that the observed signals are due to the vibrations of the electrons present in the Ag NPs and their interaction (resonance) with the electromagnetic waves of visible light; a phenomenon characteristic of metallic NPs, known as surface plasmon resonance (SPR). Both signals present similar absorption features, but the signal at ~280 nm is tapered in the UV part which indicates a high quantity of Ag NPs in the band at ~420 nm. Since the UV vis spectra are characterized by their size, shape, and distance within agglomerates [68]; a Gaussian curve was fitted to the absorption band corresponding at ~420 nm SPR in order to calculate the full width at half maximum (FWHM), see inset figure. The FWHM value is about 100.4 nm and it is similar for Ag-liposome nanocomposites to that reported by Barani et al., (78–110 nm), indicating a broad size distribution of Ag NPs [69]. Furthermore, the obtained value, which has been shown to be the maximum of the absorption band at ~420 nm, is related to the existence of nanoparticles of 48 nm average in diameter, so the synthesized silver NPs are presumably in the nanometric order [70]. 

An initial approximation of the particle size was realized by DLS measurements. The hydrodynamic radius was calculated according to the Stokes-Einstein equation; $Dh=\frac{K_{B}T}{3\pi nD_{t}}$. In this equation, *Dh* is hydrodynamic diameter; *K_B_* is Boltzmann constant, 1.3806 × 10^−23^ J/K; T is the temperature, 25 °C; n is medium viscosity with a value of 0.8878 cP, and *Dt* is the translational diffusion coefficient, which is 1.459 × 10^−8^ cm^2^ s^−1^. The hydrodynamic radius (*Dh*) obtained by DLS for the CTS-NPs Ag composite was about 126.1 ± 19.3 nm and the polydispersity Index (PDI) displayed a value of 0.250, indicating a high polydispersity [71]. This result was only considering as an estimation due to DLS to calculate the hydrodynamic radius of the Ag NPs protected with CTS polymer, whose surface can interact with the water in which the sample is suspended and thus to increase the diameter of the CTS-Ag NPs complex. Then, determination of a real particle size of the electrospun samples was determined by TEM and discussed in the next section.

**Figure 3:**
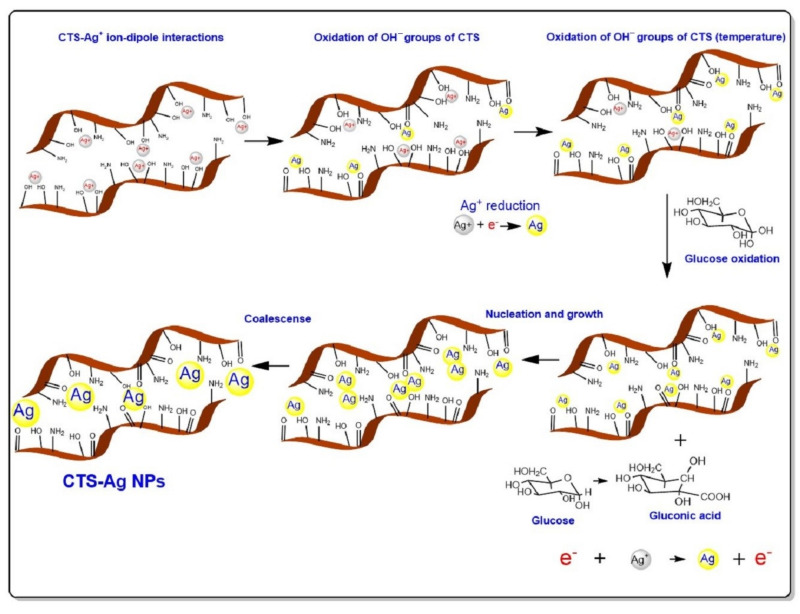
Scheme indicating the synthesis route of Ag NPs in situ CTS by chemical reduction with glucose.

Based on the FTIR, XRD and UV-Vis outcomes, a synthetic pathway to the organic-inorganic compound was proposed, as shown in Figure 3. For the CTS-Ag NPs compounds, the in situ synthesis favored the reduction process from the Ag^+^ into Ag^0^, due to the intrinsic reducing character of CTS and the reaction conditions. At the beginning of the synthesis, the AgNO_3_ salt was dissociated into Ag^+^ and ${\mathrm{NO}}_{3}^{-}$, and when it is blended with the CTS solution, the Ag^+^ ions interact electrostatically with the OH^−^ groups and the C−O−C glycosidic bond of the polymer, forming an ion-dipole interaction. This interaction is due to the excess of electrons of the oxygenated groups which favor the attraction to transition metal cations, such as silver. The reduction of silver in the presence of CTS may be due to three steps: the first one takes place before the addition of glucose as the reducing agent and it consists of the oxidation of the OH^−^ groups of CTS into carbonyl groups; as result, silver is reduced to elemental silver. The second step may be due to the high temperature of the reaction system, which could act as a catalyst for the reduction of Ag^+^ to Ag^0^ due to the action of the single electrons of the CTS oxygen that have remained unoxidized, making the reduction of Ag^+^ to Ag^0^ more complete; this would allow the formation of a monocrystalline Ag core [72]. Finally, glucose plays an important role related to the CTS reduction, promoting a third pathway. Glucose presents more active sites for the oxidation through of free aldehyde groups [57] which could donate an electron to Ag^+^ allowing its reduction to Ag^0^. The formation of Ag NPs possibly does not end with the formation of the single-crystal core, but the change in the conformation of the CTS leads to the coalescence of the single-crystal cores, forming larger particles. 

On the other hand, the shape changes and absence of the bands corresponding to the amide and amino groups of the CTS-Ag NPs with respect to pure CTS suggest that the nitrogen groups are protection sites of the Ag NPs stabilizing the hybrid compound [73,74]. Comparing the synthesis of CTS-Ag NPs with the reported synthesis of CTS-ZnO NPs, it was observed that the presence of Ag NPs allows both primary and secondary interactions, whereas in the case of ZnO NPs, only physical interactions between the NH_2_ and OH^−^ occurred. This evidence might indicate that the Ag-hybrid compound possesses superior physical properties [75].

### 3.2. Characterization of PVA-CTS and PVA-CTS-Ag NPs Electrospun Fibers

#### 3.2.1. Structural Properties

**Figure 4:**
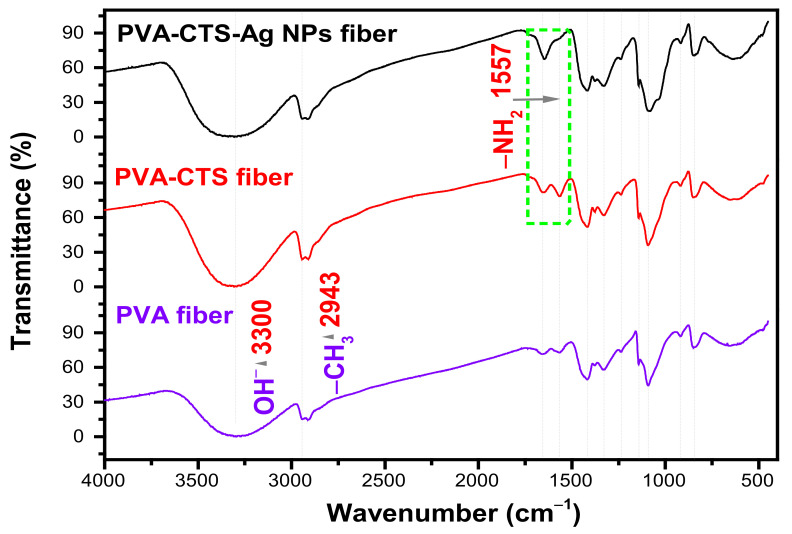
FT-IR spectra of pure PVA, PVA-CTS and PVA-CTS-Ag NPs electrospun fibers.

To confirm structural changes between the polymers PVA, CTS after addition of Ag NPs (PVA-CTS-Ag NPs), the FTIR measurements were carried out and spectra are shown in Figure 4. In this figure, also pure PVA and PVA-CTS spectra of fibers are included as a reference. In the pure PVA fiber spectrum, the characteristic stretching vibrations bands of OH^–^ at 3300 cm^−1^ and the asymmetric stretching characteristic of −CH at 2943 cm^−1^ were observed. The band corresponding to the carbonyl stretching, C=O, was identified at 1656 cm^−1^ and the band corresponding to the −CH stretching at 1566 cm^−1^. Additionally, at 1420 cm^−1^ the bending of the –CH_2_ vibration was observed in the carbonyl chain of PVA, at 1377 cm^−1^ the stretching of −CH_2_ was seen, and at 1330 cm^−1^ the bending of -CH_2_ was observed [30]. The stretching vibration of the C−O−C glycosidic bond was found at 1235 cm^−1^, while the stretching vibration of the carbonyl group, C=O, was observed at 1142 cm^−1^. The band of the C−C stretching of the acetyl group of PVA was identified at 1090 cm^−1^. Finally, the OH^−^ bending vibration in-plane was found at 919 cm^−1^ and the stretching band of CH_2_ at 850 cm^−1^. In the case of the PVA-CTS fibers, the band corresponding to the stretching of the C=O group of the PVA, (1656 cm^−1^) was acquired with higher intensity with respect to the pure PVA spectrum, which is attributed to the overlap of this band with that corresponding to the stretching of the C=O group of the CTS (1655 cm^−1^). The intensity of the band further increased in the PVA-CTS-Ag NPs spectrum, and a shift towards lower wavenumbers (blue shift) was observed at 1644 cm^−1^, suggesting interactions between the Ag NPs and the C=O groups of both polymers. In the PVA-CTS spectrum, it was found that the band at 1566 cm^−1^ corresponding to -CH increased its intensity and disappeared in the spectrum of PVA-CTS-Ag NPs. Although the formation of new absorption bands was not observed, the increased in intensity in the PVA-CTS blend is attributed to the overlap of the -CH band (1566 cm^−1^) of PVA with the band of the −CONH_2_ amide group of CTS (1557 cm^−1^) suggesting an interaction between both groups. Additionally, the intensity of the band corresponding to the stretching of the C=O of PVA with the stretching of the C=O of CTS (1655 cm^−1^) is increased, indicating PVA-CTS interactions through amide, carbonyl and/or methylene groups. Other important observations in the FT-IR spectra are the disappearance of the typical bands of amide groups of CTS and Ag NPs. Finally, in comparing the PVA-CTS and PVA-CTS-Ag NPs spectra, it can be highlighted that the band at 1377 cm^−1^ corresponding to the stretching of the -CH_2_ in PVA and the secondary stretching of the OH^−^ group in the CTS was observed to be slightly stronger in both spectra. The signals at 1420, 1330 and 850 cm^−1^ corresponding to the −CH_2_ vibrations, as well as the stretching vibration of the glycosidic bond (1090 cm^−1^), remained unchanged in PVA-CTS and PVA-CTS-Ag NPs. Thus, the observations of FTIR spectra indicated the formation of the PVA-CTS-Ag NPs compound. 

These results reveal that the functional groups of PVA do not interact significantly with Ag NPs, with the exception of the carbonyl group that is part of the acetyl group, where a slight shift (~12 cm^−1^) was observed due to the addition effect of Ag NPs. The results confirm that there exists a strong interaction between CTS and inorganic nanostructures, and this prevails after the electric field applied during the electrospinning process, which is stronger than that observed for ZnO nanostructures in similar systems [49].

#### 3.2.2. Morphological and Microstructural Analysis

**Figure 5:**
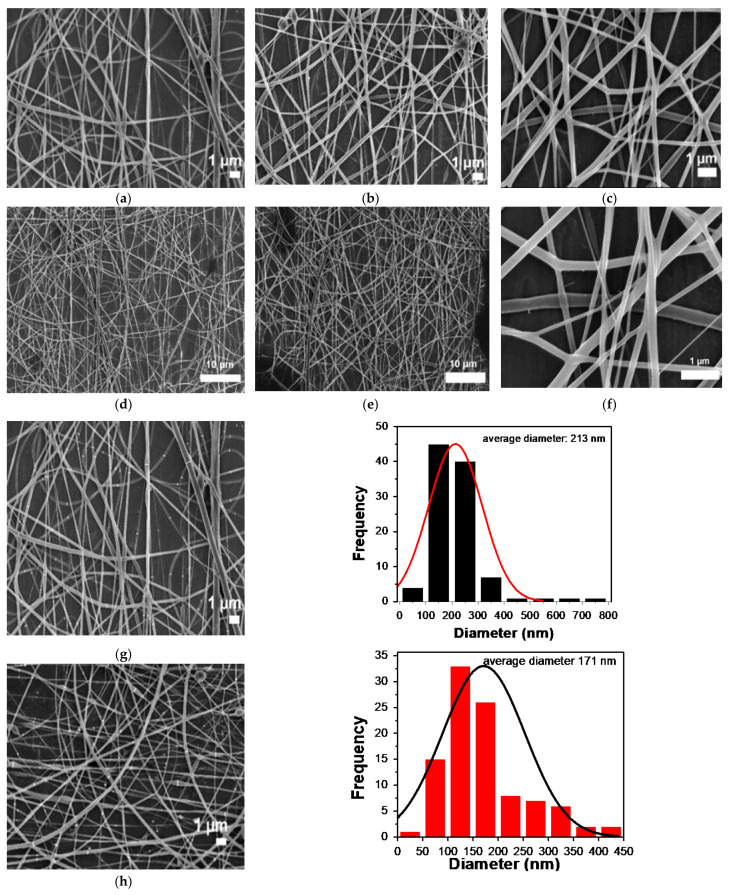
SEM images at different magnifications corresponding to (**a**–**g**) the PVA-CTS-Ag NPs electrospun fibers with its corresponding size distribution histogram. For comparison, also shown is the morphology of PVA-CTS electrospun fibers with a representative size distribution histogram (**h**).

The SEM images of PVA-CTS-Ag NPs electrospun fibers at different magnifications are shown in Figure 5a–g. The fibers presented a smooth surface with very few defects compared to the pure polymer fibers (Figure 5h). The average diameter of PVA-CTS-Ag NPs fibers was 213 nm (see histogram Figure 5g), i.e., ~42 nm larger than the average diameter of the pure polymer fibers (Figure 5h).

The increase in the average diameter of the PVA-CTS-Ag NPs fibers with respect to the PVA- CTS fibers could be attributed to an increase in viscosity above the critical value because of the increase in electrical conductivity. However, the fibers were smoother and better controlled than PVA-CTS-ZnO NPs electrospun fibers (271 nm) reported in our previous study [49]. The use of Ag NPs implies an increase in the conductivity of the composites, which leads to an increase of the applied electric field. It has been previously stated that higher viscosity can favor the electrospinning process; however, when this increase exceeds a critical value, fibers in larger diameters can be produced with **a** number of defects. In this context, the evaluated conditions of this study were adequate to obtain defect free electrospun nanofibers of the nanocomposites with a more uniform morphology compared to ZnO NPs [57].

**Figure 6:**
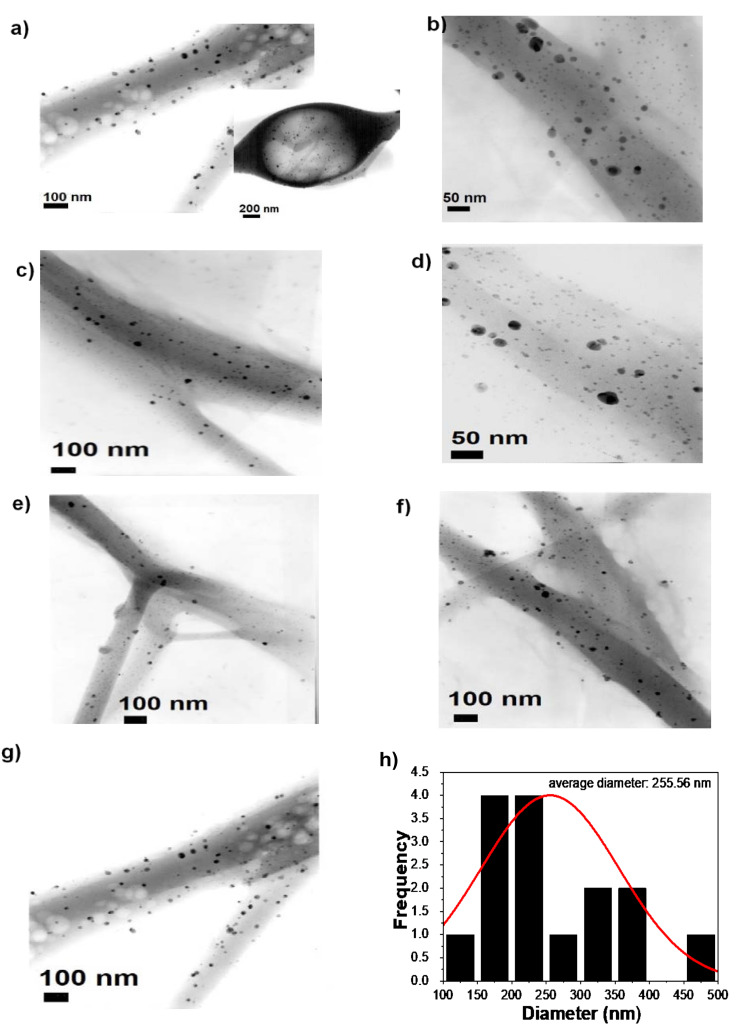
(**a**–**g**) TEM images of PVA-CTS-Ag NPs electrospun fibers and (**h**) size distribution histogram.

TEM images of the electrospun PVA-CTS-Ag NPs fibers are shown in Figure 6a–h. In agreement with the observations made by SEM, the electrospun fibers exhibited well dispersed nanostructures with smooth morphology and minimal bead-like defects (Figure 6a–g). The fibers have diameters ranging from 100 nm to 500 nm, with an average diameter of ~256 nm (Figure 6h). Additionally, Ag NPs were highly dispersed in the fibers, helping to produce almost defect-free electrospun fibers.

**Figure 7:**
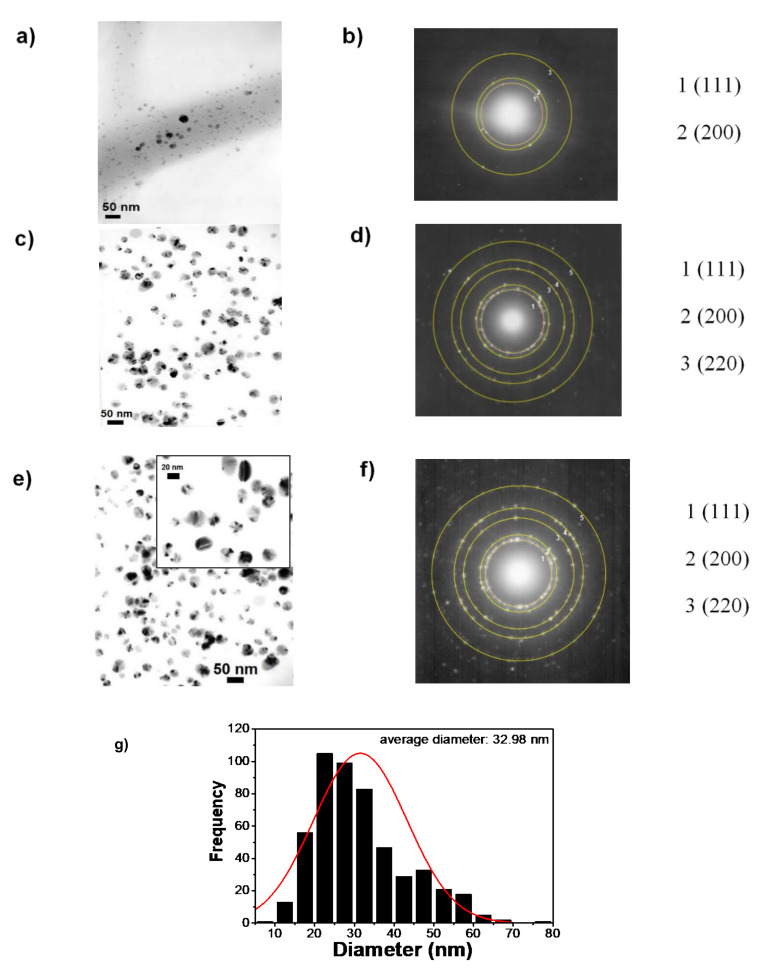
TEM images of (**a**,**b**) PVA-CTS-Ag NPs and (**c**–**f**) CTS-Ag NPs electrospun fibers with their corresponding SAED (selected area electron diffraction) patterns. (**g**) Size distribution histogram for Ag NPs in the PVA:CTS-Ag NPs compound.

In order to observe the structure type and size of Ag NPs in the PVA-CTS-Ag NPs electrospun fibers, the selected area electron diffraction (SAED) patterns were acquired (Figure 7a,b). Likewise, TEM images of the Ag NPs (Figure 7c,e) were analyzed in order to compare the diffraction ring pattern with respect to that of the electrospun fibers (Figure 7d,f). For the electrospun fibers and NPs, the interplanar distances are in agreement with those reported in the crystallographic PDF #01-089-3722 chart, corresponding to the (111), (200), (311) and (222) planes, which confirm the presence of Ag NPs with cubic structure inside the electrospun fibers. These results suggest that the formation of the electrospun fibers by the electrospinning process did not affect the structural properties of the Ag NPs, which is indicative of the high miscibility of the CTS-Ag NPs compound with the PVA solution performed before the electrospinning process. TEM images showed that Ag NPs were well-dispersed inside the fiber. From the TEM images and the average diameter analysis (counts of 500 fibers), the Ag NPs distribution was found to range from 1 to 80 nm, with an average diameter of ~33 nm for Ag NPs (Figure 7g); in addition, it is observed that more than 50% of the fibers have a size smaller than 32 nm. This distribution is in agreement with the observed dispersity in DLS measurements and previous reported studies [56]. Thus, the structure and particles size of the Ag NPs remain constant after the electrospinning process. For comparison, TEM observations showed the particle size of Ag to be a slightly larger than that computed from the Scherrer equation (14 ± 4 nm).

The mismatch can be correlated with the fact that XRD technique measures the size of coherent diffraction domains of Ag NPs; however, the actual diameter of the twinning particles have more than one diffraction domain [76]. The Ag NPs possess a grain boundary and twinning, which makes sense with the difference between XRD and TEM crystal sizes (Figure 7e).

#### 3.2.3. Mechanical Properties

**Figure 8:**
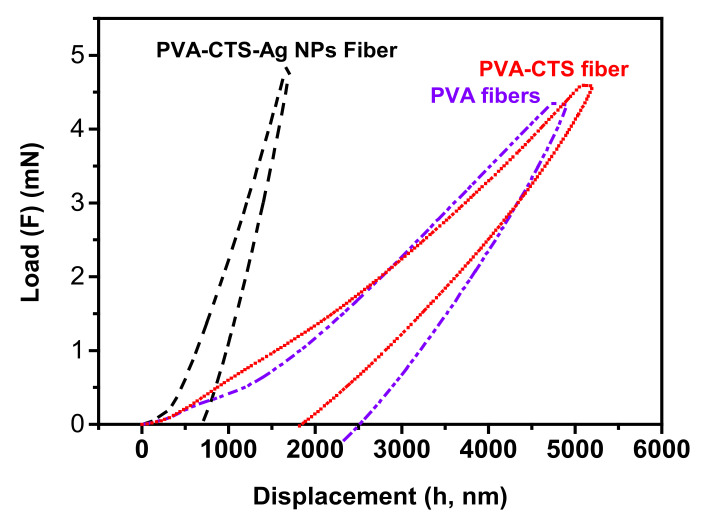
Load-displacement nanoindentation curves of PVA, PVA-CTS and PVA-CTS-Ag NPs electrospun fibers.

The mechanical properties were evaluated using the nanoindentation technique assuming that the fibers obtained were nanometric. Young´s modulus and the hardness of the PVA-CTS-Ag NPs compounds were estimated from the load vs. displacement curve of the electrospun fibers (Figure 8). The PVA and PVA-CTS fibers are also shown in the figure and the computed values are displayed in Table 1. The PVA-CTS-Ag NPs compound shows a typical curve of a soft elastoplastic polymeric material. This behavior is dissimilar to that of other composites containing ceramic nanostructures, but it maintains the trend of the pure polymers; either PVA or the combination of them (PVA-CTS). Another point to highlight is that, compared to the other systems, the penetration depth is reduced with Ag NPs incorporation (Table 1). The high values of the instrumental elastic modulus (EIT = 25 GPa) and instrumental hardness (HIT = 152 GPa), confirm a significant increase in the hardness of the fibers after Ag NPS addition. The results pointed out that the use of Ag NPS increased not only the dispersion but the morphology and mechanical properties in comparison with other works [56] or ceramic nanoparticles, particularly with ZnO and TiO_2_ NPs [49,77].

#### 3.2.4. Antibacterial Assessment

**Figure 9:**
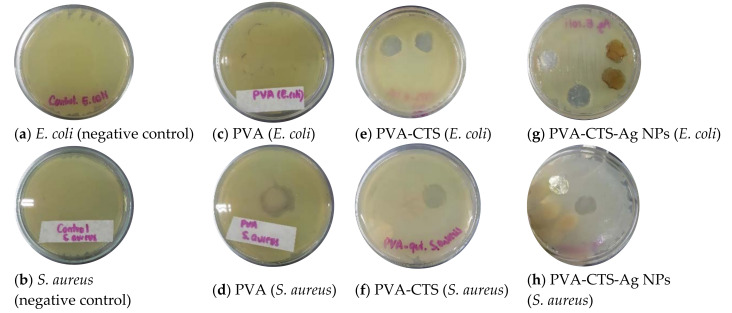
Bacterial growth of *E. coli ATCC 25922 and S. aureus ATCC 25923* in the presence of electrospun fiber mats, (**a**,**b**) negative controls, (**c**,**d**) pure PVA fibers, (**e**,**f**) PVA-CTS fibers, (**g**,**h**) PVA-CTS-Ag NPs fibers.

To complete the evaluation of PVA-CTS-Ag NPs of electrospun fibers for biomedical applications, the bactericidal effect of PVA-CTS mats and PVA-CTS-Ag NPs mats was evaluated by the Kirby-Bauer method. In addition, a pure PVA mat was evaluated to appreciate the CTS and Ag NPs contribution to bacterial inhibition. Table 1 and Figure 9a–h show the bacterial growth inhibition halos for each fiber and its comparison with other previously reported systems. Negative controls without fibers or NPs for *E. coli ATCC 25922* (0 mm) and *S. aureus ATCC 25923* (0 mm) are shown in Figure 9a,b.

PVA did not show inhibition on bacterial growth (Figure 9c,d), and in PVA-CTS fibers mats, inhibition of bacterial growth was observed below and around the sample area (Figure 9e,f), which could be attributed to the intrinsic antibacterial character of CTS due to its amino groups [80]. The PVA-CTS-Ag NPs fibers showed a typical bactericidal effect against the *E. coli ATCC 25922* (22 mm) and *S. aureus ATCC 25923* (20 mm) strains (Figure 9g,h), presenting a larger inhibition halo with respect to the PVA-CTS fibers mats (20 mm). According to Li et al., the antibacterial properties of chitosan are affected by the type of microorganism, the degree of deacetylation, molecular weight, concentration, pH value, chitosan source, temperature, cell growth phase, chitosan, and metal compound, among other factors [81]. It is generally known that the bactericidal action of chitosan is due to the permeabilization of the cell surface, which causes leakage of substances and leads to cell death. Another mechanism of bactericidal action is by affecting the cellular DNA. On the other hand, the antibacterial activity of silver can be described by different mechanisms. The first one is correlated with the fact that Ag^+^ ions react with thiol (–SH) groups present in some important enzymes inside the cell. Another mechanism is based on the weak acid nature of silver which, when reacting with phosphates in the cellular genetic material, disrupts protein translation. A third mechanism proposes that Ag^+^ ions, when in contact with the cell membrane, produce reactive oxygen species (ROS), which damages the integrity of the cell. In this context, it is known that the activity of Ag^+^ ions is higher in gram-negative bacteria, such as *E. coli*, since they have a thinner peptidoglycan layer, which increases the vulnerability of the cell to Ag^+^ ions and, therefore, the possibilities of damage and cell death [52].

The results confirmed an adequate integration of Ag NPs into the polymeric matrix promoting a higher bactericidal properties in the PVA-CTS-Ag NPs compounds. As expected, antibacterial activity of the PVA-CTS-Ag NPs electrospun fibers seems to be better than the antibacterial activity of the PVA-CTS-ZnO NPs [40,49], PVA-CTS-Cu NPs [56] electrospun fibers. Since this evaluation is based on electrostatic interactions, Ag NPs promote activity of cationic amines, improving the antimicrobial activity. This work corroborates that the in situ formation of Ag NPS into the CTS can produce electrospun fibers with controllable size and spinnability that are defect-free and that show good bactericidal activity. The proposed method is even better than other synthesis processes [56]; however, in vitro and in vivo biological evaluations are required in order to confirm the workability of the material for wound and burn treatment. 

## 4. Conclusions and Future Perspectives

In this work, PVA-CTS-Ag electrospun fibers were synthesized by employing an environmentally friendly method in order to evaluate their potential application as coating or wound dressings to avoid diverse causes of skin trauma in humans. Particularly, changes in the microstructural and mechanical properties after the electrospinning process were analyzed**.**

The main findings of this work were that the Ag NPs within the polymer matrix presented a cubic structure that was not changed during the electrospinning process, while the elastic modulus as well as the instrumental hardness, which are important properties for biomedical applications, were significantly improved with the addition of Ag nanoparticles. Additionally, it was found that Ag displayed a strong interaction with the nitrogenous compounds and the oxygens of the glycosidic bonds contained in the CTS. This interaction was related with the fact that CTS cover the Ag NPs surfaces, which also result in well-dispersed, stable NPs. The Ag NPs presented an average diameter of ~33 nm, which was maintained even after the electrospinning process, while the average diameter of the PVA-CTS-Ag NPs fibers was 213.0 nm. Finally, the electrospun PVA-CTS-Ag NPs fibers showed superior antibacterial activity to PVA-CTS and other previous reported fibers (PVA-CTS-ZnO NPs) against *E. coli and S. aureus*. This result highlights the synergy between Ag and CTS, promoting a better antibacterial character. PVA-CTS-Ag NPs fibers are viable materials for applications in the biomedical field, specifically in the form of wound and/or burn dressings; although their biological characterization needs to be completed with in vitro and in vivo assays including cytotoxicity analysis, bacterial infiltration and adhesion, healing rate, histological assessment, and bioaccumulation in order to evaluate their potential in the proposed application.

## Figures and Tables

**Table 1:** Relationship of the mechanical and antibacterial properties of the as-prepared electrospun fibers and their comparison with other metallic nanostructures.

Electrospun Nanofibers	HIT (GPa)	EIT (MPa)	Inhibition Halo		Reference
			*E. coli* (mm)	*S. aureus* (mm)	
PVA	16 ± 1.32	2.6 ± 0.36	Not active	Not active	This work
Neat PVA	0.25 × 10^−3^	-	Not active	Not active	[78]
PVA-CTS	32 ± 2.47	3.4 ± 0.40	20.0	20.0	This work
PVA-CTS	3.65 × 10^−3^ (tensile strength)	--	7.0	7.0	[79]
PVA-CTS	-	-	Less antibacterial activity than PVA-CS-Ag NPs	-	[57]
PVA-CTS	-	-	14.1	5.4	[40]
PVA-CTS	<0.05 (hardness)	100 (elastic modulus)	-	-	[77]
PVA-ZnO (5 wt%)	Larger than neat PVA (bearing load)	Smaller than neat PVA (elongation)	1	1.3	[78]
PVA-CTS-Ag NPs	152 ± 5.2	25 ± 9.88	22.0	20.0	This work
PVA-CTS-Ag NPs (0.5 wt%)	4.52 MPa (tensile strength)	-	13.3	14.3	[79]
CTS-PVA-nHA-Ag NPs			Greater antibacterial activity than CTS-PVA-nHA-Cu NPs	-	[56]
PVA-CTS-Ag NPs	-	-	Greater antibacterial activity than PVA-CTS		
PVA-CTS-ZnO NPs	42 ± 2.23	2.1 ± 0.16	20 mm	20 mm	[49]
CTS-PVA-ZnO NPs	-	-	21.8	21.5	[40]
PVA-CTS-TiO_2_ NPs	<0.025 (hardness)	170 (elastic modulus)	-	-	[77]

## Data Availability

All the data are reported in this document.
